# Biomimetic Wearable Device for Dysphagia: Digital Conceptual Design via KANO-EWM-TOPSIS and Extended FCBS Mapping

**DOI:** 10.3390/biomimetics11080548

**Published:** 2026-08-03

**Authors:** Ke Yin, Zilin Mao, Yiting Cui, Aimin Zhou

**Affiliations:** 1 College of Fine Arts, Guangdong Polytechnic Normal University, Guangzhou 510663, China; 1350000@gpnu.edu.cn (K.Y.); maomao0212@stu.gpnu.edu.cn (Z.M.); 382029260@stu.gpnu.edu.cn (Y.C.); 2 School of Architecture and Art Design, Lanzhou University of Technology, Lanzhou 730050, China

**Keywords:** dysphagia, biomimetic wearable collar, extended FCBS mapping, hybrid MCDM, KANO model, entropy weight method, TOPSIS, usability evaluation

## Abstract

Biomimetic wearable devices provide non-invasive physical support for elderly patients with dysphagia. However, most existing assistive products adopt fixed layouts designed through empirical methods and cannot meet differentiated needs in household, social, and medical scenarios. To fill this gap, this study constructed an integrated design framework combining the KANO-EWM-TOPSIS hybrid model and extended FCBS mapping. The KANO model was used to classify functional attributes and exclude mandatory safety indicators from weighting calculations to prevent data dilution. The entropy weight method (EWM) and TOPSIS were then adopted to calculate scenario weights and prioritize core functions. To resolve structural incompatibility in traditional rigid braces, a morphological biomimetic design scheme was proposed. Referring to the anatomical outline of the thyroid cartilage and movement rules of the infrahyoid muscle groups, a high-fidelity 3D digital model of the wearable collar was established. The biomimetic structure replicates the soft tissue compliance of the human neck. It offers adequate physical support without limiting vertical laryngeal displacement during swallowing. Simulated usability tests were conducted with 16 participants, and the fuzzy comprehensive evaluation (FCE) returned an overall score of 77.91. The digital design balances physiological safety and users’ psychological feelings while reducing obvious medical styling. This study offers a repeatable quantitative process and practical reference for developing human larynx anatomy-based biomimetic wearable rehabilitation devices at the preliminary design stage.

## 1. Introduction

Dysphagia is a prevalent condition in older adults, affecting 30–40% of the global population aged over 65 [1]. It weakens oral feeding capacity and raises risks of fatal aspiration pneumonia, substantially reducing seniors’ daily quality of life. Conventional rehabilitation methods, including manual therapy and dietary management, demand continuous human labor and lack real-time physiological monitoring capacity. Biomimetic wearable bioelectronic devices have developed rapidly in recent years. Distinct from rigid rehabilitation braces with uniform geometric outlines, biomimetic design replicates the biomechanical traits of biological tissues. For dysphagia intervention equipment, designers need to reproduce the flexible structure and dynamic motion paths of the human neck. The device’s exterior aligns with the anatomical shape of the thyroid cartilage. It adapts to the movement patterns of the infrahyoid muscle groups. As a result, the equipment delivers long-term, non-invasive support and continuous physiological tracking without blocking vertical laryngeal motion [2,3]. Combined with high-performance materials and sensing modules, this equipment enables personalized rehabilitation. Even so, few structured design workflows have been established to develop user-focused biomimetic wearables for elderly individuals living with dysphagia.

Current wearable devices designed for dysphagia rehabilitation mainly focus on swallowing signal sensing hardware. Most existing orthoses adopt rigid structures with simple curved surfaces, which cannot reserve enough space for vertical laryngeal movement during eating. In addition, most related products rely on designers’ subjective aesthetic adjustments rather than quantitative demand screening. Few studies combine multi-scenario user psychological needs such as social stigma into the whole design process. Traditional multi-criteria decision tools and FBS mapping are mostly applied separately, without forming a complete quantitative design workflow targeted at elderly dysphagia assistive equipment.

Biomimetic design improves human-device matching by simulating biological shapes and physiological motions, and is commonly used in joint, respiratory, and pulse rehabilitation wearables. Current swallowing-related biomimetic research mainly focuses on sensing modules [4]. Few neck devices combine laryngeal anatomy and swallowing kinematics for holistic contour optimization. Most existing biomimetic collars only use simple curved surfaces and fail to reserve space for the thyroid cartilage and infrahyoid muscle movement, which restricts natural laryngeal motion during meals. Furthermore, relevant design methods depend on subjective appearance modification rather than quantitative function evaluation, lowering the practical performance of dysphagia biomimetic wearables.

Many studies have utilized the KANO model, entropy weight method, and TOPSIS to classify product functional requirements, and the extended FBS mapping approach can translate user demands into tangible product structures [5,6]. However, most existing literature applies these methods separately, without forming a complete workflow tailored to biomimetic rehabilitation wearables. Conventional MCDM frameworks assign identical weights to mandatory safety indicators and experiential demands, which results in data dilution and biased function prioritization. Static FBS mapping fails to integrate social stigma into design logic. Other psychological factors are also excluded. These critical considerations impact elderly users’ willingness to adopt biomimetic medical wearables.

To address the above limitations, this study establishes an integrated design framework combining the KANO-EWM-TOPSIS model and extended FCBS mapping for anatomically biomimetic dysphagia collars. Unlike traditional rigid braces, the proposed equipment adopts morphological biomimetic design. The 3D digital prototype is built based on laryngeal anatomical features and swallowing kinematics, so the structure fits neck soft tissues and reserves room for vertical laryngeal motion. Based on 94 valid questionnaires collected from elderly dysphagia patients and their caregivers, the KANO model is used to classify functional attributes, and mandatory safety indicators are excluded from entropy weight calculation to avoid data dilution. EWM and TOPSIS are adopted to calculate weights for three scenarios and sort core functions by priority. After quantitative analysis under biomimetic structural constraints, a high-fidelity digital model is generated. Simulated usability tests involving 16 participants obtained an FCE score of 77.91, which proves the feasibility of this biomimetic design system.

Three key research gaps exist in current biomimetic dysphagia wearable design. First, traditional MCDM models assign equal weights to all indicators without separating mandatory safety attributes, leading to unreasonable function ranking. Second, classic FBS mapping ignores psychological constraints like social stigma and fails to convert multi-scenario demands into structural parameters. Third, most verification relies on high-cost physical prototypes, lacking low-cost digital simulation schemes for conceptual design. To fill these gaps, this paper builds an integrated KANO-EWM-TOPSIS and extended FCBS framework. Unlike prior separate method applications, we exclude must-be safety indicators from weight calculation and introduce context variables intofunction–structuremapping. The digital biomimetic collar performs well under simulated conditions and balances physical safety and user dignity. Further physical prototypes and clinical trials are needed to verify its long-term rehabilitation effect; this paper only provides a repeatable quantitative process for preliminary product development.

**Figure 1:**
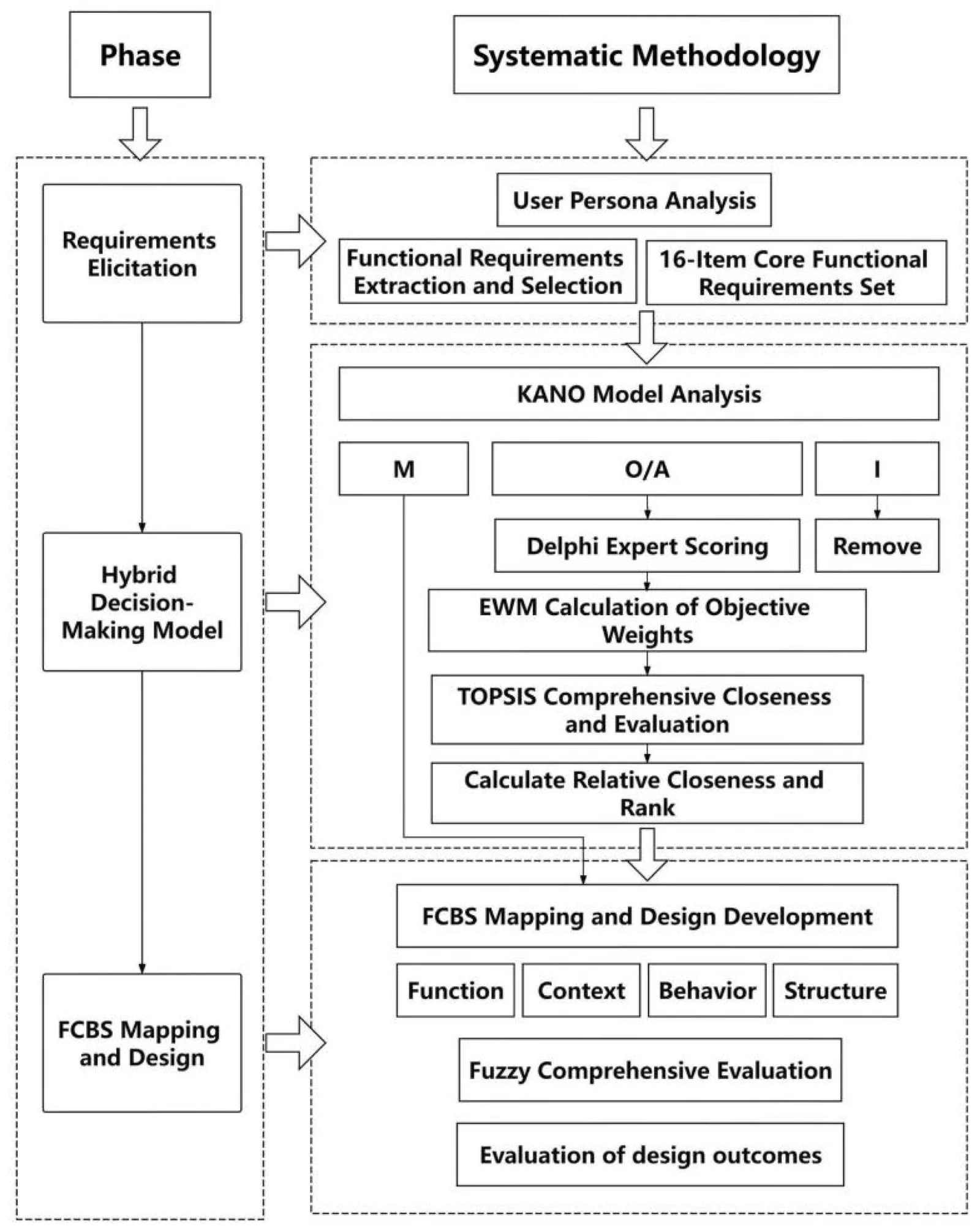
Flow Chart of KANO-EWM-TOPSIS Hybrid Decision-Making Model and FCBS Mapping Framework. Dotted rectangles denote two separate sub-modules of the proposed framework; solid arrows represent the sequential logical flow of each calculation and design step.

The rest of this paper is structured as follows. Section 2 reviews relevant theories and research methods. Section 3 details the construction of the KANO-EWM-TOPSIS multi-attribute decision model. Section 4 introduces the biomimetic structural design and usability evaluation based on FCBS mapping. Section 5 discusses research outcomes and practical design implications. Section 6 concludes this paper and proposes directions for follow-up research. Figure 1 displays the overall technical flow of the proposed biomimetic design methodology.

## 2. Literature Review and Theoretical Methods

### 2.1. Research Status of Biomimetic Wearable Rehabilitation Devices

Biomimetic design learns from the structural and functional features of organisms to develop medical rehabilitation equipment. Wearable rehabilitation devices conform to human body shapes and movement patterns, and deliver physical support for people with impaired physical functions.

Early wearable assistive devices mostly adopted rigid structures. They could realize basic auxiliary functions but fit poorly with human bodies and caused discomfort after long-time wear. With the development of biomimetics and ergonomics, flexible structures are widely applied to improve the fitting performance of wearable devices.

Most existing rehabilitation products for elderly dysphagia patients are designed for single usage scenarios. Their development mainly relies on experience and simple qualitative analysis. Systematic quantitative design methods are rarely applied, so it is difficult to balance safety, practicability, and user experience in product design. Few studies combine multi-scenario analysis and quantitative demand analysis in device development.

From a biomechanical perspective, swallowing relies on coordinated upward movement of the thyroid cartilage and contraction of infrahyoid muscle groups. Conventional rigid orthoses block such natural motion trajectories. Accordingly, biomimetic wearable design needs to mimic the elastic characteristics of human neck soft tissues, retaining enough internal clearance to support unobstructed vertical laryngeal movement while offering stable auxiliary support.

### 2.2. Research Progress of Multi-Criteria Decision-Making Methods

Multi-attribute decision-making serves as a core tool for quantitative assessment in complex product development. Traditional Multi-Criteria Decision-Making (MCDM) frameworks typically apply indiscriminate weighting to heterogeneous demands. This approach dilutes the weight of performance demands with baseline demands, causing evaluation deviations. Combining multiple models can reduce the subjectivity of single-method evaluation and improve analysis reliability in product design [7]. Furthermore, integrating these models with affective engineering resolves multidimensional psychological requirements during the morphological design phase [8].

Scholars have developed various hybrid MCDM models in recent years. Rong et al. combined the KANO model and TRIZ for product innovation design [9]. Huang et al. constructed a hybrid KANO-entropy-TOPSIS model to evaluate urban rail transit passenger service quality [10,11]. Ortíz-Barrios et al. proposed an integrated model combining the fuzzy Analytic Hierarchy Process (AHP) and TOPSIS to quantify decision-making requirements in complex industrial contexts [12]. Wang et al. used KANO-related hybrid models to reduce design bias [13]. Li et al. and You et al. combined MCDM and FBS mapping to convert demands into product structures [14,15].

Although the above methods have been widely used, they are seldom applied to elderly healthcare wearable products. Elderly rehabilitation devices need to meet strict safety standards and relieve users’ negative psychological feelings such as social stigma, which traditional MCDM methods cannot fully solve. This study constructs a KANO-EWM-TOPSIS hybrid model. The KANO model classifies functional attributes and separates safety indicators from weighting calculation to avoid data dilution. Nevertheless, quantified demands still need mapping tools to form specific product structures.

### 2.3. Evolution of Product Mapping Theory and Contextual Adaptation

The Function–Behavior–Structure FBS series models are the most widely adopted systematic mapping tools in biomimetic product design and wearable device development. They provide a fundamental framework for modern design modeling, enabling the systematic transformation of design intent into physical product structures [16]. Gero and Kannengiesser incorporated situated cognition to revise this framework, optimizing behavioral decision-making for designers in dynamic scenarios [17]. Deng et al. developed the FEBS model, which treats the environment as an intermediate variable that restricts product functions and guides behavioral modes [18].

Recent studies discuss the application of wearable devices and computer-aided screening technologies in dysphagia intervention, clarifying the importance of human-product interaction in related design work [19]. Zhang et al. proposed the FCBPSS framework by introducing context as a core ontological variable. This framework defines product function as dynamic utility expressed under specific contextual constraints. Based on context-aware technology, the team establishes mapping links between user states and system behaviors to enhance human–machine cooperation [20]. However, most traditional mapping models focus on physical environment adaptation and ignore the psychosocial constraints of elderly users. For elderly wearable medical devices, psychological experience and social perception are key factors affecting user acceptance.

For elderly patients with dysphagia, wearing medical devices in public causes strong social stigma and social isolation. Pullin proved that optimized appearance can reduce the medical label of assistive devices [21]. Many studies have confirmed the value of reducing medical styling for elderly products [22]. At present, most related work stays in qualitative discussion and subjective aesthetic adjustment. It remains a challenge to balance physiological safety and social dignity through quantitative design frameworks.

### 2.4. Summary of Literature and Research Gaps

Current studies have advanced multi-criteria decision-making algorithms and product mapping theories, providing a solid foundation for wearable rehabilitation device design. However, few studies combine quantitative design methods and the unique psychosocial needs of elderly dysphagia patients. Three key research gaps are summarized as follows:

1. Defects in demand weighting: Most hybrid MCDM models adopt unified weighting for all functional attributes. They do not distinguish between mandatory safety requirements and optional experience functions, resulting in data dilution and unreasonable function ranking.
2. Lack of context-aware mapping: Traditional mapping models only support static transformation from function to structure. They cannot reflect social and psychological factors, so it is hard to convert multi-scenario user needs into design parameters for wearable bioelectronics.
3. Insufficient digital verification means: Most design verification relies on physical prototypes, which cost much time and money. There is no systematic method to test conceptual schemes with 3D models before production, which slows down the iteration of elderly assistive devices.

To fill the above gaps, this study integrates quantitative demand screening, context-aware mapping and dignity-oriented design, and proposes an integrated framework combining KANO-EWM-TOPSIS and extended FCBS mapping for dysphagia rehabilitation wearable devices.

## 3. Construction of KANO-EWM-TOPSIS Multi-Attribute Decision-Making Model

### 3.1. Research on Contextualized User Requirements

Common MCDM combinations such as AHP-TOPSIS, ANP and BWM have clear limitations in evaluating layered rehabilitation demands. AHP and ANP rely heavily on subjective pairwise expert comparison with strict consistency tests. BWM cannot separate mandatory safety indicators from experiential functions. To address these defects, this paper integrates KANO, EWM and TOPSIS. The KANO model classifies demand attributes. EWM calculates weights based on raw data to cut subjective bias. TOPSIS ranks functions across multiple scenarios. This combination fits the evaluation of dysphagia rehabilitation wearables better than single or alternative MCDM methods.

User requirement analysis is the core of user-centered design. All questionnaire items were summarized from rehabilitation literature and clinical interviews. A pilot test was conducted to revise ambiguous items prior to formal research, and the questionnaire achieved acceptable construct validity with a Cronbach’s α of 0.722. All participants were screened by strict inclusion and exclusion criteria for mild-to-moderate elderly dysphagia patients, yielding 94 valid KANO questionnaires alongside 20 semi-structured interview records. Ten evaluators participated in Delphi scoring, including six clinical rehabilitation physicians and four senior geriatric care managers. All indicators were assessed using a unified five-point Likert scale in line with standard Delphi procedures. K-means cluster analysis (k = 4, determined via the elbow method) was adopted to categorize users, generating four typical user personas listed in Table 1 that cover different age groups and illness severities.

**Figure 2:**
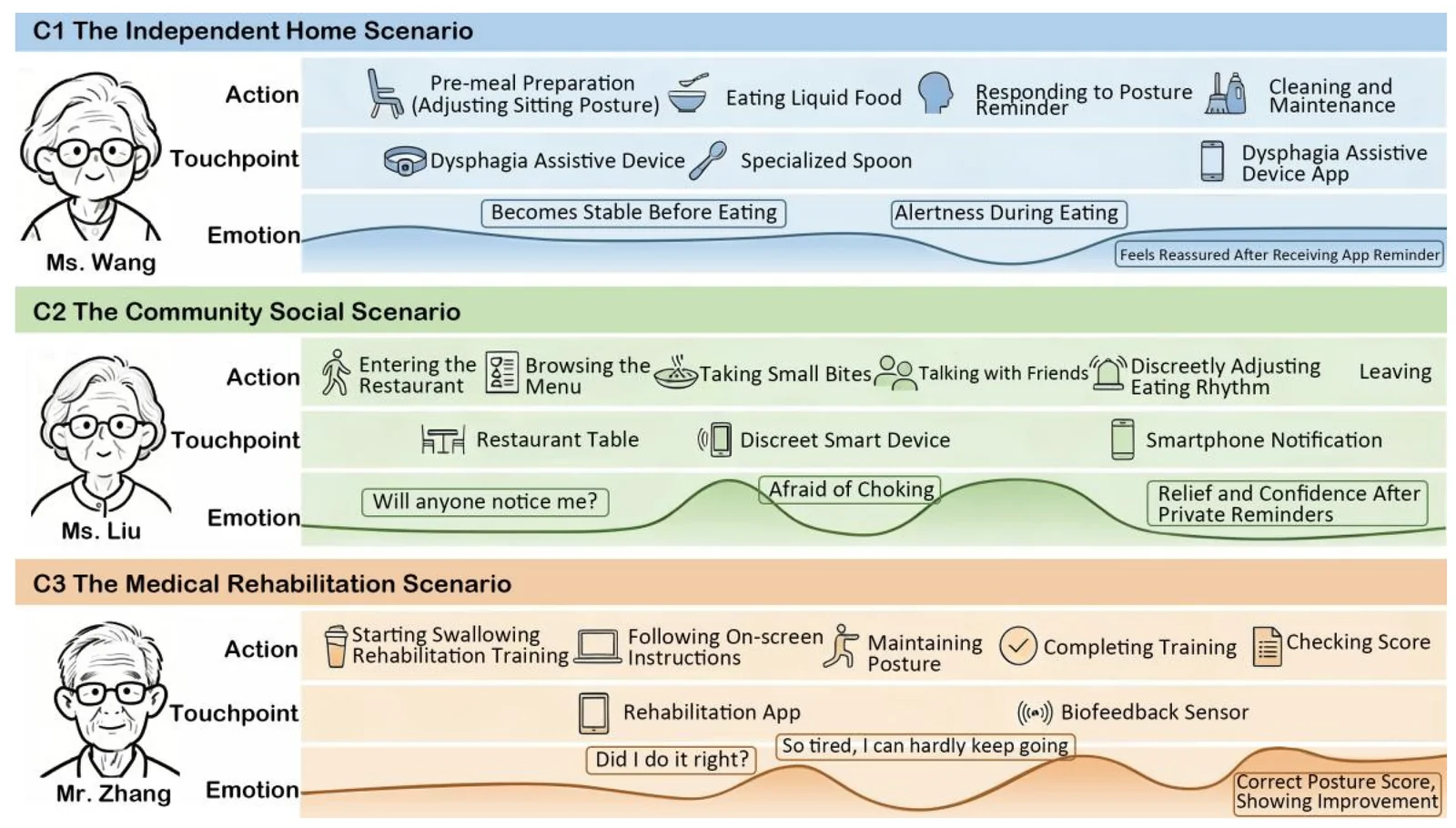
Multi-scenario User Journey Map.

User journey analysis was conducted to analyze changing demands in different scenarios. The results show that user requirements differ obviously in independent home, community social, and medical rehabilitation scenarios (Figure 2). The home scenario focuses on safety protection; the social scenario pays more attention to psychological comfort and social dignity; the medical scenario takes rehabilitation training and data feedback as the priority.

**Figure 3:**
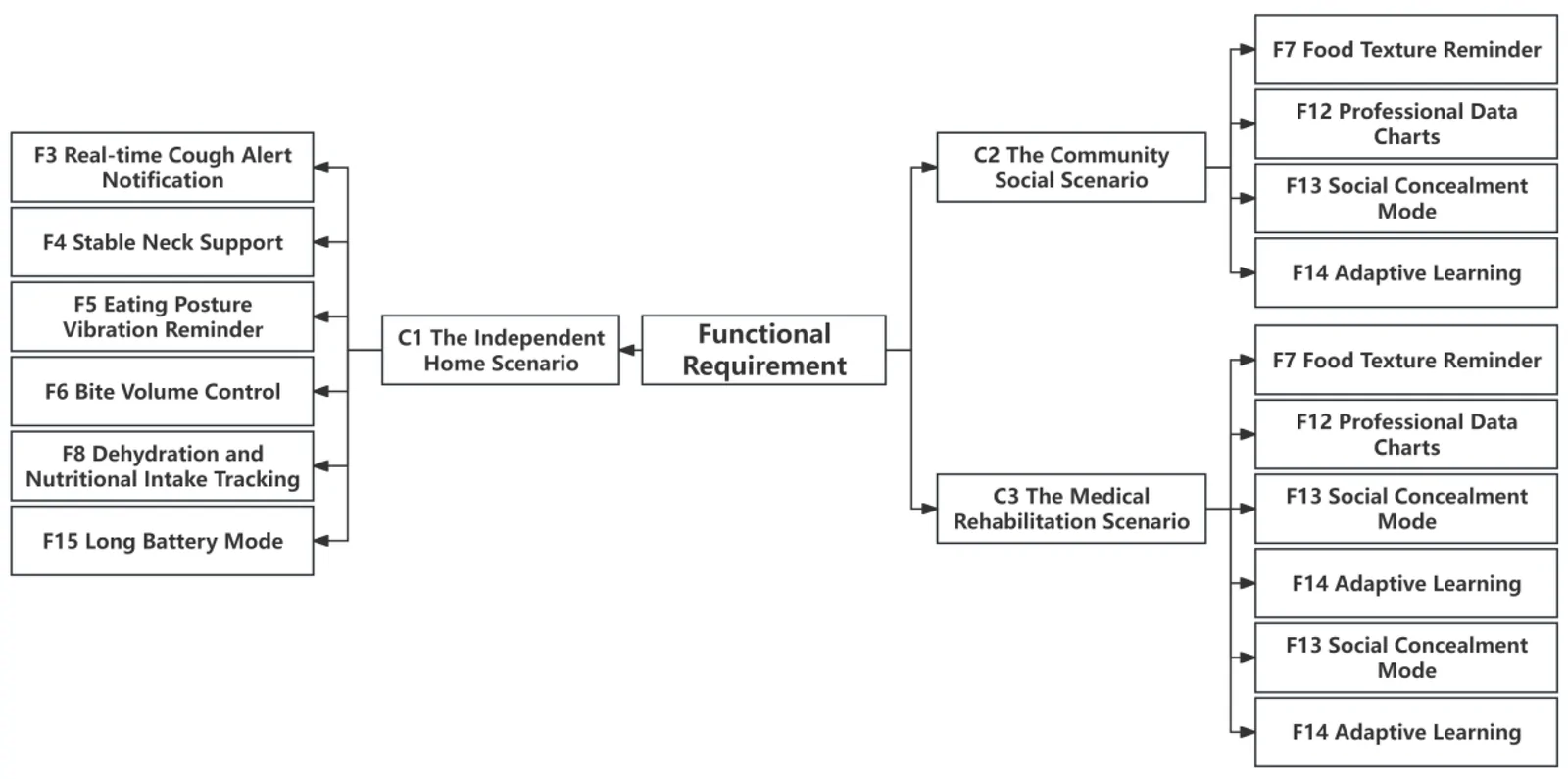
User functional requirements for biomimetic wearable dysphagia assistive devices.

Combining literature research, field investigation and user journey analysis, this study established a complete functional requirement set. A total of 16 core functional indicators were extracted, including physiological safety, functional performance, and user experience (Figure 3). These indicators provide a basis for subsequent quantitative analysis.

### 3.2. Requirement Identification and Classification Based on the KANO Model

This study collected 94 valid questionnaire responses from patients with mild-to-moderate dysphagia and their caregivers. The KANO model was used to categorize 16 functional requirements, and the Better-Worse indices provided quantitative analysis. This method distinguishes physiological safety requirements from functions related to social dignity, laying an objective foundation for subsequent weight calculation across different scenarios, as shown in Table 2. The collected 94 valid questionnaires passed basic reliability tests, and the classification results are credible for subsequent analysis.

Data analysis indicated that *F*_3_ (Real-time Cough Alert Notification) and *F*_4_ (Stable Neck Support) constituted Must-be attributes (*M*). Their dissatisfaction indices reached 79.12% and 69.2% respectively, while the corresponding satisfaction indices remained at low levels. These results confirm that physiological safety acts as a basic and rigid constraint across all usage scenarios.

*F*_13_ (Social Concealment Mode) and *F*_14_ (Adaptive Learning) were classified as attractive attributes *(A*). Both items featured satisfaction indices higher than 71% and absolute dissatisfaction indices no greater than 30%. This result verified the initial assumption that concealed interaction and adaptive functions provide positive psychological experiences and improve user social acceptance.

Indicators including *F*_1,_ *F*_5,_ and *F*_8_ were classified as one-dimensional attributes (*O*). Their bidirectional indices maintained high values, reflecting users’ clear demands for accurate monitoring and fluent interaction. By comparison, *F*_12_ (Professional Data Charts) exerted limited influence on user experience. Consequently, the model classified it as an indifferent attribute (*I*) and excluded it from further research.

The screening process retained 15 core functional indicators. These indicators entered the subsequent multi-scenario weight calculation via the entropy weight method (EWM).

### 3.3. Objective Weight Assignment via Entropy Weight Method

After KANO-based requirement classification, the Must-be attributes (*F*_3_, *F*_4_) and the indifferent attribute (*F*_12_) were excluded from weight calculation, leaving 13 functional indicators for multi-scenario objective weight assignment. Traditional subjective weighting methods are prone to personal bias. Therefore, this study adopted the entropy weight method (EWM), an objective weighting technique that determines weights based on data dispersion characteristics.

#### 3.3.1. Construction of the Decision Matrix

In this study, 10 professional experts, including 6 clinical rehabilitation physicians and 4 senior care administrators, were invited to conduct evaluations. All 13 functions were scored via a 5-point Likert scale under three typical scenarios: private home use (*C*_1_), community social scenarios (*C*_2_), and medical rehabilitation scenarios (*C*_3_).

Let $x_{ij}$ represent the average score of the i-th function under the j-th scenario. The initial decision matrix X is constructed as follows:

(1) $$X=\left[\begin{array}{ccc} x_{1,1} & x_{1,2} & x_{1,3}\\ x_{2,1} & x_{2,2} & x_{2,3}\\ \vdots & \vdots & \vdots \\ x_{13,1} & x_{13,2} & x_{13,3} \end{array}\right]$$

To unify data dimensions, the original matrix was standardized. The feature proportion $p_{ij}$ of the i-th function in the j-th scenario is calculated by:

(2) $$p_{ij}=\frac{x_{ij}}{\sum_{i=1}^{m}x_{ij}}$$

**Figure 4:**
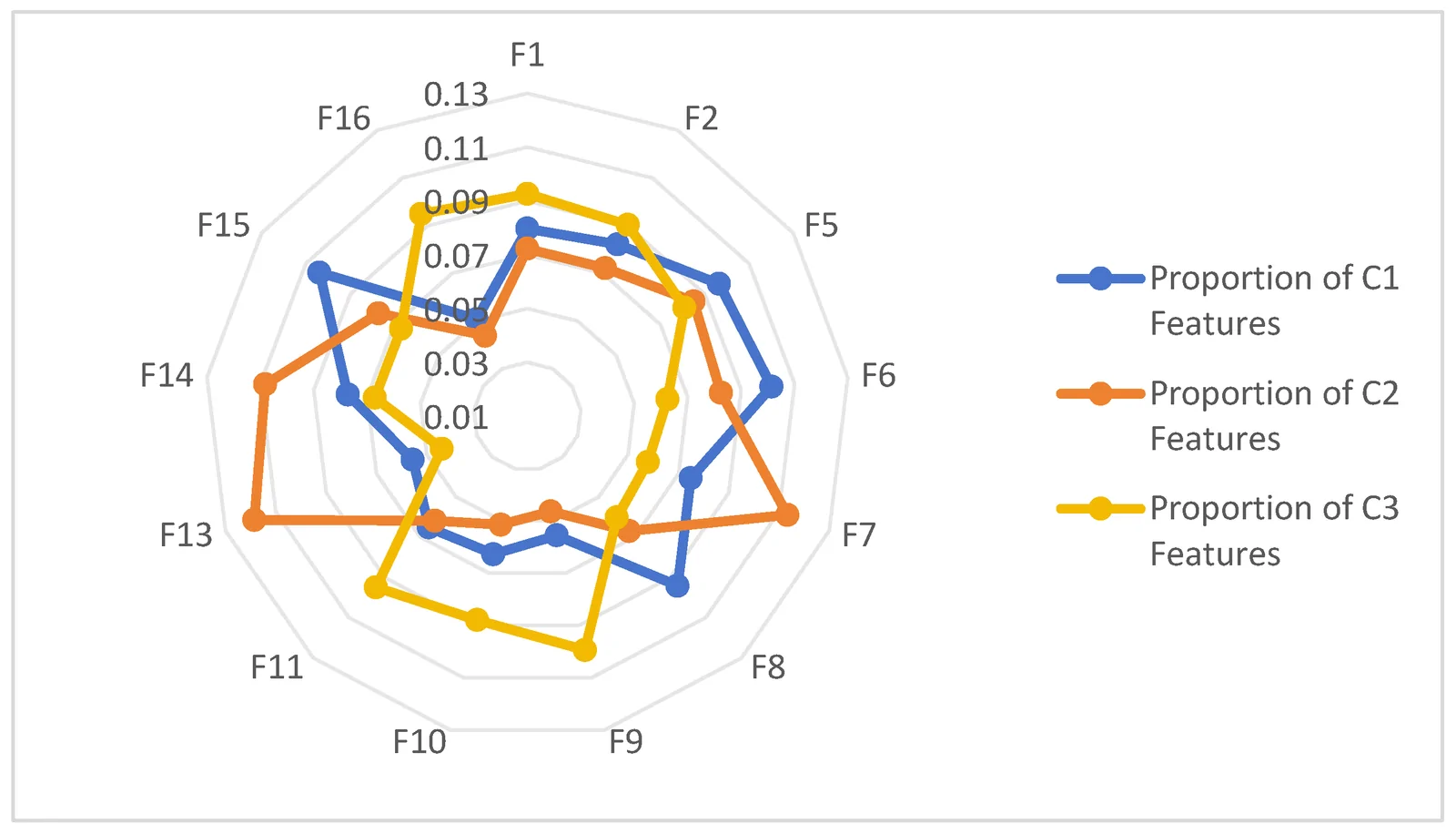
Radar chart of the multi-scenario feature weight distribution.

The original average scores and the standardized feature proportions are detailed in Table 3 and illustrated in Figure 4.

#### 3.3.2. Calculation Procedures

Based on the standardized proportion matrix *P*, we sequentially compute the information entropy $e_{j}$, difference coefficient $d_{j}$, and objective weight $w_{j}$ for each scenario. The detailed steps are presented below.

First, calculate the information entropy $e_{j}$. A larger entropy value means a smaller difference among data and less effective information:

(3) $$e_{j}=-k\sum_{i=1}^{m}p_{ij}\ln(p_{ij})$$

where m=13 denotes the total number of functional indicators, and $k=\frac{1}{\ln(m)}$.

Second, calculate the difference coefficient $d_{j}$:

(4) $$d_{j}=1-e_{j}$$

Third, normalize the difference coefficients to obtain the final objective weight $w_{j}$:

(5) $$w_{j}=\frac{d_{j}}{\sum_{j=1}^{n}d_{j}}$$

where n=3 represents the number of application scenarios. The computed weight results are summarized in Table 4.

Mandatory safety functions related to swallowing risk are removed from entropy weight calculation. This avoids data dilution caused by mixing rigid safety limits with experiential indicators and improves the objectivity of subsequent TOPSIS ranking.

The 10 participating experts consist of six clinical rehabilitation practitioners and four geriatric care managers, all with over five years of relevant working experience. Kendall’s W test confirms consistent scoring. Similar MCDM research usually employs 8–15 evaluators, and 10 experts meet the sample demand in this study.

The weight distribution results in Table 4 present significant scenario-based differences, which further verifies the rationality of introducing a multi-scenario weighting mechanism into intelligent assistive device design. According to the calculation results, the community social scenario has the highest weight, indicating user demands in public environments deserve more attention in design. The data indicates that in public social environments, elderly users have pronounced psychological demands for “stigma elimination” and “private interaction”. Therefore, this dimension is the core variable that determines the functional priority of assistive devices.

Furthermore, the weight distributions of *C*_1_ (The Independent Home Scenario, $w_{1}=0.2656$) and *C*_3_ (The Medical Rehabilitation Scenario, $w_{3}=0.2646$) are relatively balanced with small data fluctuations. This indicates that in private or professional environments without external observation, physical assistance such as aspiration prevention and rehabilitation interventions are regarded more as common basic needs of users. In conclusion, the objective weight vector obtained in this stage will be directly used as the basic input parameter for the subsequent TOPSIS comprehensive evaluation model.

### 3.4. TOPSIS Comprehensive Ranking

After obtaining objective weights for each scenario via the entropy weight method, this study adopts the TOPSIS model to evaluate the comprehensive performance of 13 functional indicators. The combination of EWM and TOPSIS forms a reliable hybrid evaluation framework for multi-scenario wearable product analysis [23]. The model calculates the relative closeness of each function to positive and negative ideal solutions to determine the priority of core functional modules [24].

#### 3.4.1. Construction of the TOPSIS Evaluation Model

The original decision matrix is normalized to establish the standardized matrix $R=(r_{ij}{)}_{13\times 3}$, and the calculation formula is shown in Equation (6):

(6) $$r_{ij}=\frac{x_{ij}}{\sqrt{\sum_{i=1}^{m}x_{ij}^{2}}}$$

where $x_{ij}$ refers to the average expert score listed in Table 3, and *m* is the number of functional indicators.

Combined with the scenario weight vector obtained earlier, the weighted normalized decision matrix *V* is constructed using Equation (7):

(7) $$v_{ij}=w_{j}\cdot r_{ij}$$

Based on matrix *V*, the positive ideal solution $V^{+}$ and negative ideal solution $V^{-}$ of 13 functions under three scenarios are defined:

(8) $$V^{+}=(v_{1}^{+},v_{2}^{+},\ldots ,v_{n}^{+}),{\;v}_{j}^{+}={max}_{i}(v_{ij})$$

(9) $$V^{-}=(v_{1}^{-},v_{2}^{-},\ldots ,v_{n}^{-}),v_{j}^{-}={min}_{i}(v_{ij})$$

The Euclidean distance from each functional item to the positive and negative ideal solutions is calculated respectively:

(10) $$D_{i}^{+}=\sqrt{\sum_{j=1}^{n}(v_{ij}-v_{j}^{+}{)}^{2}},\;D_{i}^{-}=\sqrt{\sum_{j=1}^{n}(v_{ij}-v_{j}^{-}{)}^{2}}$$

The comprehensive relative closeness of each function is calculated by Equation (11). A value closer to 1 means better overall performance across multiple scenarios:

(11) $$C_{i}=\frac{D_{i}^{-}}{D_{i}^{+}+D_{i}^{-}}$$

The distance values and final ranking results of all functions are summarized in Table 5.

#### 3.4.2. Result Analysis

According to the relative closeness values in Table 5, the 13 functional indicators were divided into three tiers to define the development priority.

The first tier ($C_{i}>0.60$) includes *F*_14_ (Adaptive Learning), *F*_7_ (Food Texture Reminder) and *F*_5_ (Eating Posture Vibration Reminder). These functions achieve high comprehensive scores in the evaluation. Among them, *F*_14_ ranks first, indicating that adaptive interaction capability is the core of the intelligent assistive device. *F*_7_ and *F*_5_ mainly focus on swallowing safety intervention, which are essential functions for home and rehabilitation scenarios.

The second tier ($0.40<C_{i}\leq 0.60$) contains seven functions: *F*_13_, *F*_6_, *F*_15_, *F*_2_, *F*_1_, *F*_8_ and *F*_11_. *F*_13_ (Social Concealment Mode) ranks highest in this group. It achieves a high score in the community social scenario but performs moderately in home and rehabilitation environments. This feature reflects users’ strong demand for low-visibility design in public occasions, which helps reduce psychological barriers caused by medical attributes. The rest of the functions in this tier serve as conventional modules for physical monitoring and intervention.

The third tier ($C_{i}\leq 0.40$) covers *F*_9_, *F*_10_ and *F*_16_. Their overall contribution to real-time eating intervention is relatively low. Considering users’ cognitive load and system redundancy, these modules are set as low-priority items.

Combining the KANO attribute classification and TOPSIS ranking results, this study formulates a two-level screening strategy: taking KANO Must-be attributes as rigid design constraints, and selecting high-priority functions from the first and second tiers. Finally, the core function set is determined as:

$$F_{core}=\{F_{3},F_{4},F_{1},F_{2},F_{5},F_{6},F_{7},{F_{8},F_{11},F}_{13},F_{14},F_{15}\}$$

This process completes the conversion from diverse user demands to engineering design indicators. On the premise of guaranteeing swallowing safety, it clarifies the design focus on adaptive interaction and de-medicalized features, and provides objective support for the subsequent FCBS mapping design.

Furthermore, all prioritized core functions need to comply with the spatial limits derived from anatomical bionics to avoid geometric interference in subsequent structural design.

We carry out sensitivity analysis to test TOPSIS ranking robustness. Each functional weight rises and falls by 10%. After recalculating closeness coefficients, the core function ranking remains unchanged. Only minor order shifts appear in low-priority auxiliary functions, which proves our weighting and ranking results are stable.

## 4. Practice and Validation of Intelligent Assistive Device Design Based on FCBS Mapping

This chapter presents the digital design process of the biomimetic wearable dysphagia aid using the extended FCBS mapping model. It first introduces the context-aware FCBS mapping framework, then details the structural design of the wearable collar, and finally verifies the design concept through a digital usability evaluation. The proposed method avoids the need for early-stage physical prototypes, reducing development costs and shortening iteration cycles.

### 4.1. Construction of the FCBS Model and Its Mapping Relationship with Biomimetic Wearable Assistive Device for Dysphagia

Traditional FBS models primarily focus on static function-to-structure transformations. To address the multi-scenario and psychosocial requirements of elderly users, this study extended the FBS model by adding a context (*C*) layer, forming the Function–Context–Behavior–Structure (FCBS) mapping framework [18,20].

The FCBS model contains four functional layers. The core function layer adopted the quantitative results from the hybrid decision model and defined the basic tasks to meet user demands. The usage context layer includes environmental factors, social norms, and psychological constraints such as social stigma. This research determined three typical application scenarios: the independent home scenario (*C*_1_), the community social scenario (*C*_2_), and the medical rehabilitation scenario (*C*_3_). The interactive behavior layer sets corresponding operation strategies for different contexts, converting complex dynamic adaptation into regular interactive modes. This design facilitates human–machine collaboration by identifying implicit user intentions rather than relying on explicit manual commands. The entity structure layer provides hardware and morphological support for all interactive behaviors. This layer applies de-medicalized design to align the product appearance with social norms. Table 6 presents the detailed definitions of each layer.

**Figure 5:**
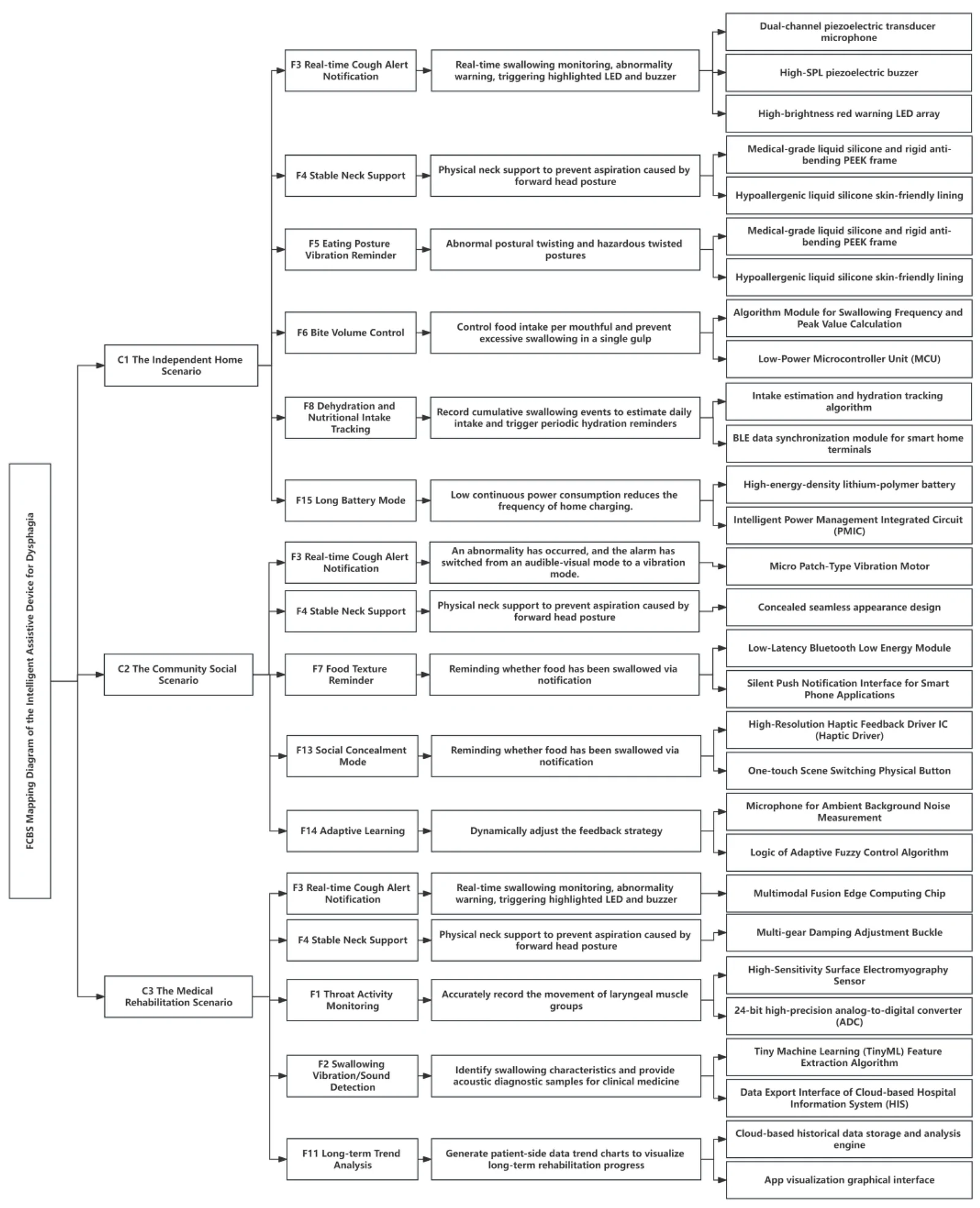
FCBS multi-dimensional scenario mapping framework.

To maintain system stability and lower operation difficulty for elderly users, the FCBS framework adopts a modular integration scheme. Following the priority ranking from TOPSIS results, the system groups core functions into independent clusters. Users switch between different scenarios via a one-touch operation. This design reduces sensor false triggers and eases the cognitive burden on users. The context variable *C* acts as an environmental trigger to drive the microcontroller unit (MCU) and adjust system behaviors (*B*), as shown in Figure 5 and Table 7.

When the working mode switches from *C*_1_ to *C*_2_, the control system executes feedback suppression. It disables high-power acoustic outputs, activates low-latency Bluetooth Low Energy (BLE) modules, and transmits warning signals to micro-tactile components via high-resolution haptic driver chips. This working mode optimizes hardware resource allocation and prevents sensory misoperation.

The context variable *C* establishes boundary rules for the entire mapping process. When the system activates *F*_13_ (Social Concealment Mode) under social scenarios, it disables sound and light alarms and utilizes micro-tactile units for silent feedback. Concurrently, the MCU adapts to ambient noise, and the BLE module transmits food-related reminders to the paired mobile terminal.

In this mapping framework, morphological biomimetic principles serve as rigid structural boundaries for the Structure (S) layer. All interaction behaviors and hardware layouts derived from Function–Context logic must comply with anatomical and kinematic limits of the human neck.

### 4.2. Design Practice of Biomimetic Wearable Assistive Device for Dysphagia

#### 4.2.1. Ergonomic Dimension Design

The design strictly followed ergonomic principles across three dimensions: dimensional optimization, shape design, and material selection. A de-medicalized appearance reduces users’ psychological resistance to wearable medical equipment. The product shape fits the anatomical features of the thyroid cartilage and infrahyoid muscle groups.

To address neck muscle atrophy and cervical forward bending prevalent in elderly groups, this study constructs a dimensional correction model referring to China’s national standard (GB/T 10000-2023) for adult human body dimensions [25]. All dimensional parameters of the biomimetic collar are derived from standardized national anthropometric data together with published swallowing biomechanical research, which supplies quantitative anatomical foundations for the wearable device’s fitting performance. Specifically, the digital design sets the horizontal inner diameter of the neck ring to 130.0 mm to prevent compression against the carotid artery. It compresses the height of the anterior avoidance area to 48.0 mm to ensure normal vertical laryngeal movement during swallowing. A 35.0 mm adjustable range at the posterolateral section adapts to users with different body shapes and wearing tightness demands. Table 8 presents the detailed ergonomic parameters and correction bases.

#### 4.2.2. Form and Material Design

Based on the dimensional calibration, the neck collar adopts a continuous curved wrapping structure with rounded front corners. This structure reserves space for the thyroid cartilage and ensures close contact between the internal sensors and the skin. To address the weakened hand strength and poor fine motor ability of elderly users, the solution incorporates magnetic connections with large clearances for all detachable parts. This mechanism enables users to independently don, doff, and clean the device, preserving self-care dignity while maintaining physiological monitoring continuity.

**Figure 6:**
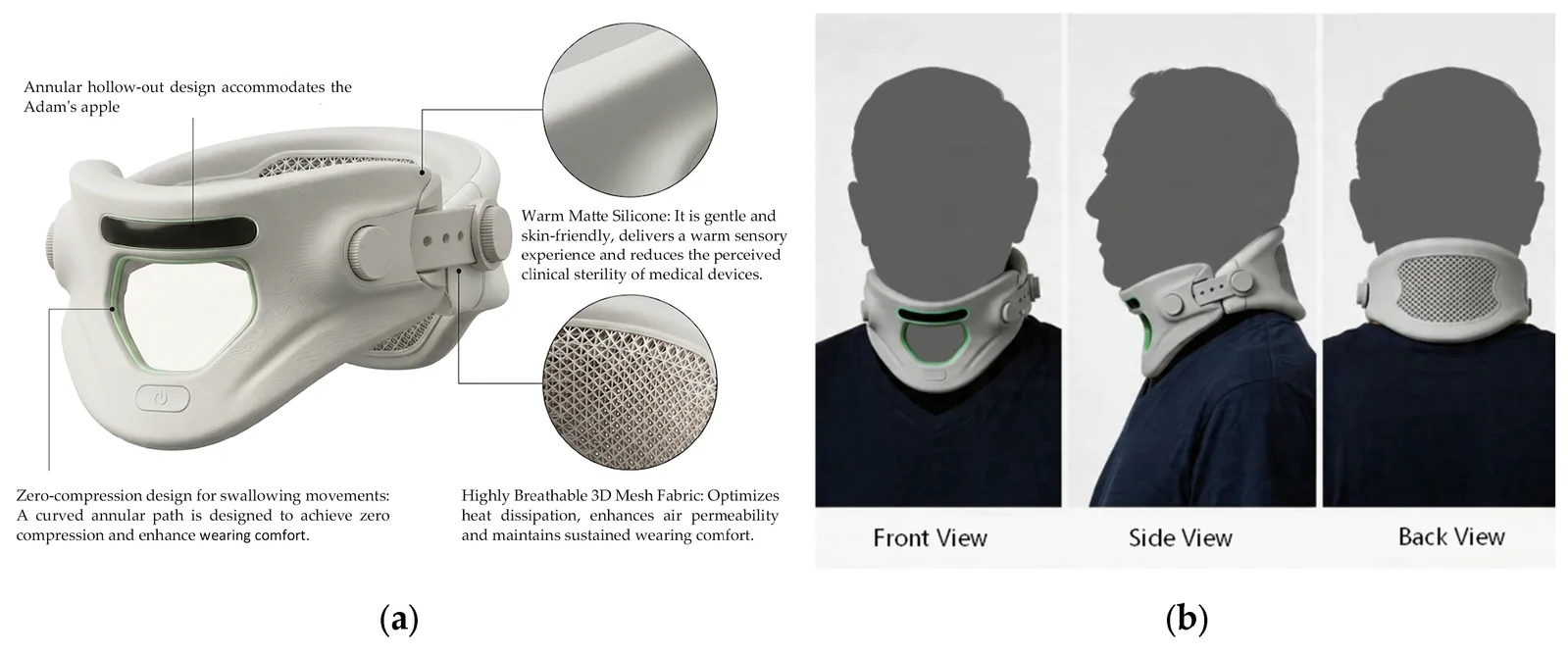
(**a**) 3D rendering of the proposed device. (**b**) Three-view drawing of the worn collar.

To accommodate the skin of the elderly, the device utilizes a multi-layer composite structure. The inner contact layer consists of hypoallergenic medical liquid silicone to improve skin compatibility and wearing comfort. Combining external non-invasive flexible sensing technology with an ergonomic design, the device captures the kinematic characteristics of the larynx and classifies swallowing movements [26,27]. The outer supporting frame comprises lightweight PEEK (polyether ether ketone) material, providing anti-drop impact resistance. The product incorporates an IPX6-grade hydrophobic nano-coating and a low-saturation ivory gray tone to weaken the medical visual semantics. Figure 6 presents the overall rendering and three-view drawings of the device.

#### 4.2.3. Biomimetic Features and Functional Improvements

The proposed wearable collar incorporates three biomimetic design features derived from laryngeal anatomy and swallowing kinematics. These features address the poor fit and motion restriction of traditional rigid braces. Table 9 outlines their biological references and functional enhancements.

These features are implemented through specific engineering designs:

1. The inner surface is calibrated against national anthropometric standards (GB/T 10000-2023) to match neck morphology.
2. The multi-layer base pairs a medical-grade silicone inner layer with a lightweight PEEK outer frame to form gradient stiffness.
3. The 48 mm anterior clearance reserves vertical space for laryngeal elevation during swallowing.

These design details align with the ergonomic parameters established earlier in this section.

### 4.3. System Feasibility Verification Based on Fuzzy Comprehensive Evaluation

To verify the feasibility of the proposed design, a simulated usability test was conducted using high-fidelity 3D digital models. The Fuzzy Comprehensive Evaluation (FCE) method was adopted to quantitatively assess the overall performance of the biomimetic wearable collar.

#### 4.3.1. Evaluation System Design

The evaluation system consisted of three layers: an objective layer (A), a criterion layer (*B*), and an indicator layer (*C*), which directly correspond to the three typical application scenarios defined in Section 3:

1. Physiological safety reliability (*B*_1_): Targets the independent home scenario (*C*_1_), focusing on baseline risk intervention performance.
2. Social dignity maintenance (*B*_2_): Corresponds to the community social scenario (*C*_2_), evaluating the effectiveness of de-medicalized appearance design.
3. Medical collaboration and training effectiveness (*B*_3_): Maps to the medical rehabilitation scenario (*C*_3_), assessing data visualization quality and training compliance.

A 5-point Likert scale was used to establish the comment set *V* = {excellent, good, fair, poor, very poor}, with corresponding quantitative values assigned for defuzzification.

#### 4.3.2. Experimental Setup

A total of 16 participants (7 males, 9 females) aged 60–75 years with mild-to-moderate dysphagia were recruited for the test. This sample size is consistent with standard practices in formative usability testing. Previous human factors engineering studies indicate that 10 to 15 participants are typically sufficient to identify major interaction issues in conceptual prototypes [23].

#### 4.3.3. Data Processing and Results

Reliability analysis of the collected questionnaires yielded a Cronbach’s α coefficient of 0.722, indicating acceptable internal consistency of the survey data. The weights of the three primary dimensions were determined using the entropy weight method (EWM) results from Section 3, with equidistant weighting applied to secondary indicators within each dimension.

Membership degrees of all secondary indicators were calculated through frequency statistics and data normalization. The resulting fuzzy evaluation matrix is presented in Table 10.

After obtaining the evaluation results of each subsystem, the model introduces the first-level indicator weight matrix *W*_A_ = [0.2656, 0.4697, 0.2646]. The overall membership matrix *R* comprises the vectors [$B_{1}$, $B_{2}$, $B_{3}$]^T^. The system implements the weighted operation as follows:

(12) $$B_{total}=W_{A}\cdot R=W_{A}\cdot \left[\begin{array}{c} B_{1}\\ B_{2}\\ B_{3} \end{array}\right]$$

The calculation determined the overall evaluation vector:

$$B_{total}=[0.2722,0.4199,0.2390,0.0689,0.0000]$$

A 100-point assignment vector *S* = [100, 80, 60, 40, 20]*^T^* enables the quantitative calculation based on the weighted average rule:

(13) $$Score=B_{total}\cdot S=77.91$$

The result indicates that the overall performance of the proposed design falls within the “good” range. The evaluation scores are concentrated in the “good” (41.99%) and “excellent” (27.22%) categories, confirming the engineering feasibility and user acceptance of the de-medicalized appearance and adaptive vibration interaction design.

## 5. Discussion

### 5.1. Core Research Findings

The fuzzy comprehensive evaluation (FCE) results showed that the proposed 3D digital model-based conceptual design achieved an overall usability score of 77.91, corresponding to the “good” evaluation grade. According to the TOPSIS ranking results, Adaptive Learning (*F*_14_) and Social Concealment Mode (*F*_13_) ranked among the top four functional modules. This indicates that context-aware interaction is as important as physical anatomical support for elderly dysphagia users.

Traditional assistive devices often use exposed mechanical structures for fitting adjustments, which may cause psychological distress and social stigma, leading to device abandonment. In contrast, the proposed design used micro-tactile actuators to output localized vibration pulses instead of high-volume acoustic alerts, implementing a covert interaction logic. This design meets elderly users’ privacy needs in simulated public environments while maintaining baseline swallowing safety constraints observed in digital tests. The decent overall evaluation score partly benefits from the neck collar’s anatomical biomimetic contour that avoids interfering with swallowing motions.

### 5.2. Comparative Analysis with Traditional Symmetrical MCDM Baselines

To verify the performance of the proposed KANO-EWM-TOPSIS framework, this study benchmarked it against three mainstream multi-criteria decision-making (MCDM) methods: KANO-AHP [24], traditional EWM-TOPSIS [28], and Fuzzy AHP-TOPSIS [29]. The same dataset (94 user responses and 10 expert ratings) was used for all methods to ensure a consistent baseline. Five evaluation metrics were selected: social scenario weight (*C*_2_), FCE usability score, inverted cognitive load index, safety balance, and experience priority.

Conventional MCDM methods apply indiscriminate weighting to all functional indicators. This dilutes the statistical weight of psychosocial demands, often resulting in over-designed physical structures and poor user compliance. Additionally, some hybrid models rely heavily on expert scoring, introducing subjective bias into ranking results. Unlike these approaches, the proposed method excludes must-be safety indicators from the entropy weight calculation matrix. This eliminates interference from rigid constraint items and improves the accuracy of cross-scenario demand assessment.

**Figure 7:**
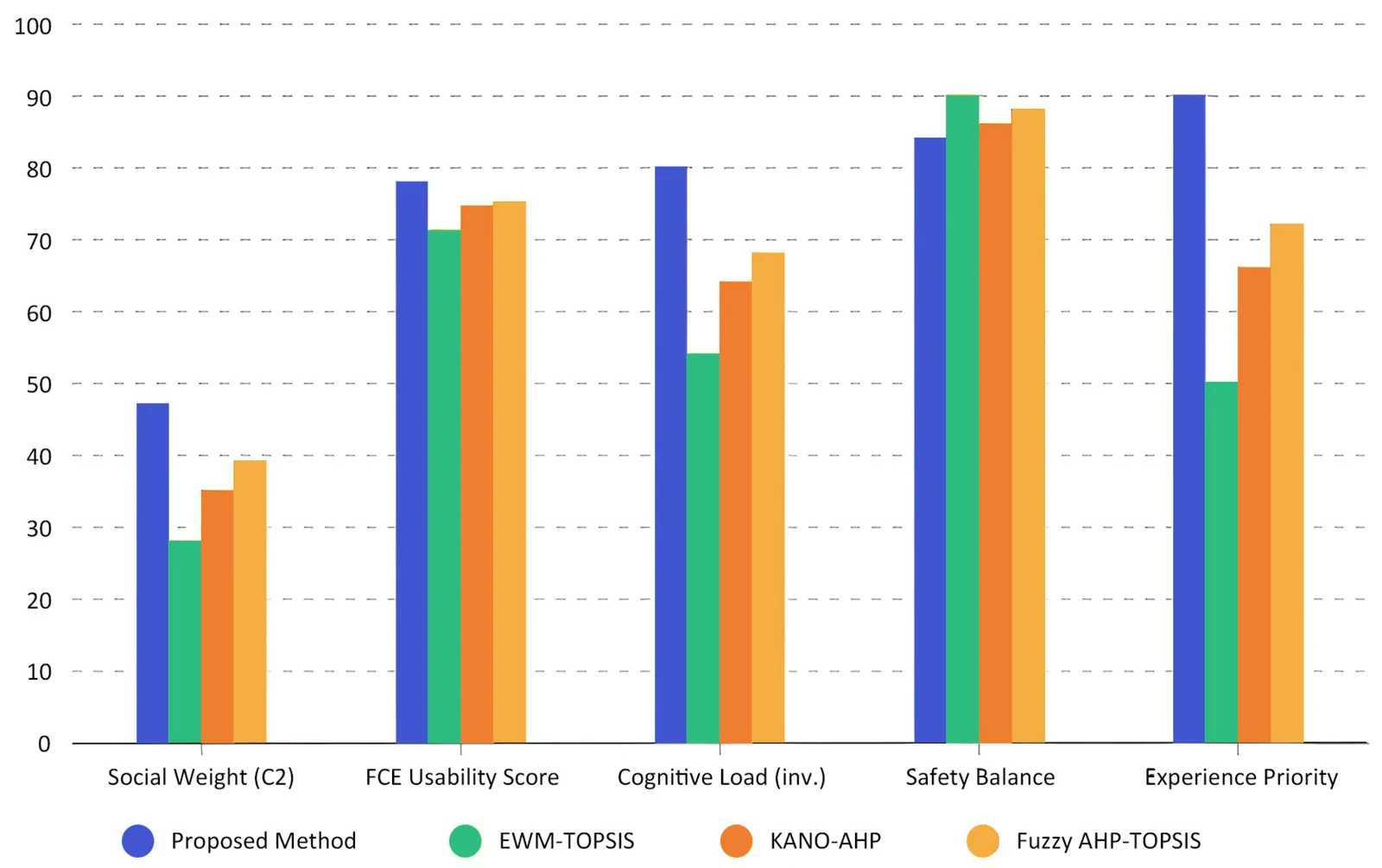
Multi-dimensional performance comparison between the proposed method and three benchmark MCDM methods. All indicators are normalized based on Table 11, and the cognitive load indicator has been inverted (a higher value of this indicator indicates better performance).

Table 11 and Figure 7 summarize the quantitative results. Compared with other methods, the proposed framework achieves a higher social scenario weight and better usability score. Furthermore, since design evaluation is conducted using 3D digital models rather than physical prototypes, functional configuration conflicts are resolved at the conceptual phase, avoiding late-stage structural iterations. This data-driven approach achieves a more reasonable balance between physiological safety and user experience, reducing early-stage development costs of wearable bioelectronics. Conventional weighting methods ignore the geometric limits of biomimetic wearable structures, leading to unreasonable function allocation.

### 5.3. Social Sustainability and Inclusive Design

The proposed FCBS mapping framework provides a structured reference for the inclusive development of elderly-oriented assistive products. Clinical observations show that swallowing disorders and corresponding interventions often lead to social isolation and psychological distress in elderly patients [30]. To address this, the proposed design implements a de-medicalized appearance and covert tactile feedback. This reduces prominent medical visual features, allowing users to participate in daily social activities more comfortably.

This approach applies inclusive design principles throughout the early conceptual stage. The 3D digital models balance physical rehabilitation requirements with user psychosocial experiences. In the home (*C*_1_) and medical rehabilitation (*C*_3_) scenarios, the system maintains necessary structural constraints and uses LED displays for visual data tracking. In the public social scenario (*C*_2_), the control system switches to silent micro-tactile feedback. This confirms that data-driven functional screening can improve human–machine compatibility for vulnerable user groups before physical manufacturing. The de-medicalized visual effect is realized through the biomimetic structural design matching human neck morphology.

### 5.4. Practical Implications and Design Inspirations

The hybrid decision framework and FCBS mapping model provide quantitative references for the design of rehabilitation wearable devices. According to the FCE results, 6.89% of negative feedback comes from complex operational workflows. Complex interactive functions may cause psychological anxiety in elderly users with reduced cognitive abilities.

For subsequent product iterations, designers should prioritize unobtrusive passive monitoring modes to reduce user cognitive load. Additionally, functional modules *F*_10_ and *F*_11_ enable data interconnection between home monitoring terminals and cloud-based medical platforms. This supports the deployment of medical Internet of Things (mIoT) systems and provides a data-driven paradigm for building integrated community elderly care networks.

This quantitative demand screening framework has general applicability. After adjusting evaluation indicators and questionnaire items, it can be applied to limb rehabilitation equipment. Targeted revision of constraint conditions is required for severe or young dysphagia groups.

### 5.5. Limitations and Future Research Directions

This study has three main limitations. First, the usability test sample size of 16 participants limits the generalizability of the findings. Second, the current digital scheme lacks optimized signal denoising and recognition algorithms for complex environments. Third, the framework has only been validated for dysphagia assistive device designs.

All usability tests in this work rely on digital models rather than physical prototypes. Digital simulation cannot fully reflect real wearing pressure, material friction and long-term wearing comfort. Physical prototypes and real-user trials are required to further test the collar’s practical performance.

This study only verifies the framework on mild and moderate elderly dysphagia users. More tests on other crowds and devices are needed to expand its application scope.

Follow-up research will produce physical prototypes based on this digital model. We will adopt sEMG measurement and laryngeal kinematic tracking to conduct real-wear tests, to analyze the biomechanical interaction between the brace and human neck soft tissues.

Future research will conduct larger-scale user tests using digital simulations to improve statistical robustness. Context-adaptive signal processing algorithms will be optimized for noisy scenarios. Furthermore, extending this framework to other elderly assistive device designs will help establish a universal design paradigm for wearable products in connected healthcare networks.

## 6. Conclusions

This study presented an integrated design framework that combines KANO-EWM-TOPSIS multi-criteria analysis and extended FCBS mapping for anatomically biomimetic dysphagia collars. This workflow excludes mandatory safety indicators from weight calculation and adopts morphological biomimetic rules to construct a high-fidelity 3D neck model conforming to laryngeal movement characteristics. Usability evaluation based on digital simulation yields an overall FCE score of 77.91, showing good performance in simulated tests. This study offers quantitative design references for digital conceptual design of rehabilitation wearables, and physical prototypes are needed to verify its actual rehabilitation effect. Future work will increase the sample size of user tests, optimize context-aware signal processing algorithms, and extend this framework to other senior assistive product designs.

## Figures and Tables

**Table 1:** Representative User Personas for Dysphagia.

User	Basic Info	Physical Condition	User Needs
Mr. Zhang	68, urban resident, retired middle school teacher, mild dysphagia	Occasional choking, in peak recovery period	Comfortable with digital health data; seeks rehabilitation guidance and real-time feedback from device
Ms. Li	65, urban resident, retired clerical worker, moderate dysphagia	Needs food texture adjustment; severe eating-related stigma	Enjoys socializing but fears public choking; needs discreet alerts to preserve social dignity
Ms. Wang	75, town resident, early-stage high-age functional decline, moderate dysphagia	Muscle loss reduces swallowing ability; long-term care by spouse	Concerned about safe eating; needs easy-to-use device that monitors intake and alerts for dehydration
Mr. Zhao	82, rural resident, elderly with multiple chronic diseases, high choking risk	Delayed swallowing reflex, relies on semi-liquid diet, difficulty using digital devices	Needs device with automatic monitoring and emergency alerts to ensure safety when alone

**Table 2:** Summary of KANO attribute classification and coefficients for functional requirements.

Serial Number	Functional Requirement	A	O	M	I	R	Q	Attribute	$S_{i}$	$D_{i}$
*F*_1_	Throat Activity Monitoring	22	31	18	21	1	1	O	57.61%	−53.26%
*F*_2_	Swallowing Vibration/Sound Detection	20	44	13	15	2	0	O	69.57%	−61.96%
*F*_3_	Real-time Cough Alert Notification	12	10	62	7	2	1	M	24.18%	−79.12%
*F*_4_	Stable Neck Support	9	8	55	19	1	2	M	18.68%	−69.23%
*F*_5_	Eating Posture Vibration Reminder	17	48	16	10	1	2	O	71.43%	−70.33%
*F*_6_	Bite Volume Control	11	37	32	12	2	0	O	52.17%	−75.00%
*F*_7_	Food Texture Reminder	52	7	14	19	1	1	A	64.13%	−22.83%
*F*_8_	Dehydration and Nutritional Intake Tracking	16	68	5	3	1	1	O	91.30%	−79.35%
*F*_9_	Rehabilitation Training Guidance	63	12	8	9	2	0	A	81.52%	−21.74%
*F*_10_	Clinical Data Export	14	41	29	8	0	2	O	59.78%	−76.09%
*F*_11_	Long-term Trend Analysis	34	46	6	7	0	1	O	86.02%	−55.91%
*F*_12_	Professional Data Charts	14	5	11	58	3	3	I	21.59%	−18.18%
*F*_13_	Social Concealment Mode	75	4	9	5	1	0	A	84.95%	−13.98%
*F*_14_	Adaptive Learning	49	15	12	14	2	2	A	71.11%	−30.00%
*F*_15_	Long Battery Mode	28	34	19	11	1	1	O	67.39%	−57.61%
*F*_16_	Gamified Incentive	57	21	10	4	1	1	A	84.78%	−33.70%

**Table 3:** Initial Decision Matrix and Feature Weight Distribution.

Serial Number	Functional Requirement	$x_{i1}$	$p_{i1}$	$x_{i2}$	$p_{i2}$	$x_{i3}$	$p_{i3}$
*F*_1_	Throat Activity Monitoring	3.3	0.0797	2.8	0.0722	4.0	0.0926
*F*_2_	Swallowing Vibration/Sound Detection	3.4	0.0821	2.8	0.0722	3.9	0.0903
*F*_5_	Eating Posture Vibration Reminder	4.0	0.0966	3.3	0.0851	3.5	0.0810
*F*_6_	Bite Volume Control	4.2	0.1014	3.2	0.0825	2.7	0.0625
*F*_7_	Food Texture Reminder	3.1	0.0749	4.4	0.1134	2.5	0.0579
*F*_8_	Dehydration and Nutritional Intake Tracking	3.9	0.0942	2.6	0.0670	2.6	0.0602
*F*_9_	Rehabilitation Training Guidance	2.3	0.0556	1.8	0.0464	4.3	0.0995
*F*_10_	Clinical Data Export	2.6	0.0628	2.0	0.0515	3.8	0.0880
*F*_11_	Long-term Trend Analysis	2.7	0.0652	2.4	0.0619	4.1	0.0949
*F*_13_	Social Concealment Mode	2.3	0.0556	4.6	0.1186	1.9	0.0440
*F*_14_	Adaptive Learning	3.2	0.0773	4.2	0.1082	2.9	0.0671
*F*_15_	Long Battery Mode	4.3	0.1039	3.0	0.0773	2.9	0.0671
*F*_16_	Gamified Incentive	2.1	0.0507	1.7	0.0438	4.1	0.0949
Total	-	41.4	1.0000	38.8	1.0000	43.2	1.0000

**Table 4:** Entropy weight calculation results for three major application scenarios.

Evaluation Criterion (Scenario)	$e_{j}$	$d_{j}$	$w_{j}$
*C*_1_ The Independent Home Scenario	0.9898	0.0102	0.2656
*C*_2_ The Community Social Scenario	0.9820	0.0180	0.4697
*C*_3_ The Medical Rehabilitation Scenario	0.9898	0.0102	0.2646

**Table 5:** Positive and Negative Ideal Solutions and Relative Closeness Coefficients.

Item	Distance to Positive Ideal Solution ($D_{i}^{+}$)	Distance to Negative Ideal Solution ($D_{i}^{-}$)	Relative Closeness ($C_{i}$)	Ranking
*F*_14_ Adaptive Learning	0.080	0.165	0.674	1
*F*_7_ Food Texture Reminder	0.093	0.172	0.650	2
*F*_5_ Eating Posture Vibration Reminder	0.087	0.145	0.624	3
*F*_13_ Social Concealment Mode	0.133	0.177	0.570	4
*F*_6_ Bite Volume Control	0.109	0.134	0.552	5
*F*_15_ Long Battery Mode	0.114	0.132	0.536	6
*F*_2_ Swallowing Vibration/Sound Detection	0.118	0.121	0.508	7
*F*_1_ Throat Activity Monitoring	0.119	0.122	0.508	8
*F*_8_ Dehydration and Nutritional Intake Tracking	0.142	0.100	0.414	9
*F*_11_ Long-term Trend Analysis	0.151	0.105	0.409	10
*F*_9_ Rehabilitation Training Guidance	0.192	0.101	0.345	11
*F*_10_ Clinical Data Export	0.176	0.085	0.324	12
*F*_16_ Gamified Incentive	0.201	0.092	0.314	13

**Table 6:** Definition of each dimension of the FCBS model.

Dimension	Formal Symbol	Meaning and Constraint Content
Function	*F*	The set of meta-tasks that the system must perform to satisfy the user’s core goal.
Context	*C*	The physical space, social norms, and psychological constraints, such as stigma, involved in product use.
Behavior	*B*	The interaction strategy adopted by the system in response to a given context.
Structure	*S*	The physical carrier and technical support through which the behavior is implemented.

**Table 7:** Preset modes of biomimetic wearable dysphagia assistive device.

Preset Mode (*C*)	Core Function Set (*F*)	Typical Behavioral Strategy (*B*)	Structural Feature (*S*)	Key Constraint Response
Home Independent Mode	*F*_3_, *F*_4_, *F*_5_, *F*_6_, *F*_15_, *F*_8_	Strong intervention and multi-channel feedback	Voice broadcast + highlighted LED reminder	Physiological safety assurance
Social Community Mode	*F*_3_, *F*_4_, *F*_14_, *F*_7_, *F*_13_	Weak intervention and de-labeling feedback	Neck skin pulse vibration	Mitigation of social stigma
Medical Rehabilitation Mode	*F*_3_, *F*_4_, *F*_2_, *F*_1_, *F*_11_	Active guidance and data-driven motivation	App visualization interface + touch interaction	Improvement of compliance

**Table 8:** Ergonomic design parameters and correction basis for the wearable collar.

Key Part	National Standard Reference Value (mm)	Correction Value (mm)	Final Specification (mm)	Correction Basis
Horizontal Inner Diameter of Neck Ring	118.2 (P50)	+12.0 (clothing allowance)	130.0	Allows space for clothing and prevents a sense of restraint during long-term wearing.
Height of Anterior Clearance Area	62.5 (mean neck length)	−14.5 (mobility correction)	48.0	Ensures that the vertical displacement of the thyroid cartilage during swallowing is not obstructed.
Width of Sensing Window	32.0 (hyoid region width)	+18.0 (deviation allowance)	50.0	Fully covers the optimal sensing area for laryngeal electromyographic and acoustic signal acquisition.
Posterolateral Adjustment Range	N/A	0–35.0	35.0	Adapts to individualized tightness requirements for users with different body types.
Ring Cross-sectional Thickness	N/A	8.0–12.0	10.0	Balances the structural rigidity of the PC frame with lightweight wearing comfort.

**Table 9:** Mapping biomimetic features to functional enhancements *.

Concrete Biomimetic Feature	Biological Reference	Functional Improvement
Anatomical curved fitting surface	Natural contour of anterior neck soft tissue	Improves fitting accuracy; reduces slippage
Gradient flexible base	Neck skin and muscle tissue elasticity	Reduces foreign body sensation; accommodates tissue displacement
Anterior vertical clearance	Laryngeal elevation trajectory	Avoids mechanical obstruction; preserves swallowing kinematics

* Note: All biological references in this table belong to human neck anatomical tissues related to swallowing, and no animal organisms are used as bionic prototypes.

**Table 10:** Fuzzy evaluation matrix for the proposed design *.

	Weight	Secondary Indicator	Weight	Excellent	Good	Fair	Poor	Very Poor
(*U*_1_) Home Independent Mode	0.2656	Posture Reminder	0.500	0.3750	0.2500	0.3125	0.0625	0.0000
		Bite Volume Control	0.500	0.3750	0.3125	0.3125	0.0000	0.0000
		First-level Evaluation Set (*B*_1_)		0.3750	0.2813	0.3125	0.0313	0.0000
(*U*_2_) Social Community Mode	0.4697	De-medicalized Design	0.500	0.3750	0.3750	0.1875	0.0625	0.0000
		Adaptive Vibration	0.500	0.1250	0.4375	0.3125	0.1250	0.0000
		First-level Evaluation Set (*B*_2_)		0.2500	0.4063	0.2500	0.0938	0.0000
(*U*_3_) Medical Rehabilitation Mode	0.2646	Laryngeal Activity Monitoring	0.333	0.0625	0.5625	0.2500	0.1250	0.0000
		Patient-side Data Trend Chart	0.333	0.3125	0.4375	0.1875	0.0625	0.0000
		Physician-side Data Value	0.334	0.2500	0.7500	0.0000	0.0000	0.0000
		First-level Evaluation Set (*B*_3_)		0.2083	0.5833	0.1458	0.0625	0.0000

* Note: The data value indicators for the physician side were only evaluated by 4 invited senior clinical rehabilitation physicians.

**Table 11:** Quantitative Comparison with State-of-the-Art MCDM Methods.

Metric	Proposed	EWM–TOPSIS	KANO–AHP	Fuzzy AHP–TOPSIS
Social Weight (*C*_2_)	0.47	0.28	0.35	0.39
FCE Score	77.91	71.25	74.60	75.10
Cognitive Load (inv.)	4.00	2.70	3.20	3.40
Safety Balance	4.20	4.50	4.30	4.40
Experience Priority	4.50	2.50	3.30	3.60

## Data Availability

All data generated or analyzed during this study are included in this article. The raw data are not publicly available to protect the privacy of elderly participants. Readers may contact the corresponding author for reasonable data requests.
